# What is the carbon footprint of reverse osmosis in water treatment plants? A systematic review protocol

**DOI:** 10.1186/s13750-023-00316-z

**Published:** 2023-11-14

**Authors:** Samaneh Abolli, Esfandiar Ghordouei Milan, Parnia Bashardoust, Mahmood Alimohammadi

**Affiliations:** 1 Department of Environmental Health Engineering, School of Public Health, Tehran University of Medical Sciences, Tehran, Iran; 2 Student’s Scientific Research Center, Tehran University of Medical Sciences, Tehran, Iran; 3 Center for Water Quality Research, Institute for Environmental Research, Tehran University of Medical Sciences, Tehran, Iran; 4 Health Equity Research Center (HERC), Tehran University of Medical Sciences, Tehran, Iran

**Keywords:** Water, Water treatment plant, Reverse osmosis, Desalination, Carbon footprint, CO_2_ emission

## Abstract

**Background:**

“Carbon footprint” (CF) is a direct measure of greenhouse gas emissions caused by a defined activity and can demonstrate global warming effects. The emissions of Greenhouse gases (GHGs) in water projects start from the primary water sources, followed by transportation, construction, and operation phases in the final treatment plants. Due to their possible environmental impacts, the water treatment plants equipped with Reverse Osmosis (RO) units will be investigated for their carbon footprint.

**Methods:**

The research question is “What is the carbon footprint of reverse osmosis in water treatment plants?”. The literature search in this study will be divided into two sequential sections; in the first section, the search will be limited to Scopus, Science Direct, EMBASE, and PubMed databases. The keywords of water, “water treatment plants”, “water purification”, desalination, “reverse osmosis”, RO, “carbon emission”, “carbon dioxide/CO_2_ emission”, “carbon footprint”, “Life Cycle Assessment” and, LCA will be used. The carbon footprint of RO will be expressed based on the direct and indirect effects based on RO capacity. In the second section, the internet and specialist search will be done, and the search will be updated. No date limitation will be considered, and the main search will be done in English. When the search is completed, the screening will be performed. After removing duplicates, the title and abstract will be examined. The full text will be read if the title and abstract are not helpful for decision-making. In addition, the bibliography and references will proceed after the full-text screening. The Collaboration for Environmental Evidence (CEE) Critical Appraisal Tool will be used for risk of bias checking and study validity assessment. After full-text evaluation, data will be collected and categorized by two authors. If there is enough data, meta-analysis will be performed.

*Systematic review registration* PROSPERO CRD42022327572.

**Supplementary Information:**

The online version contains supplementary material available at 10.1186/s13750-023-00316-z.

## Background

Urbanization and population growth have forced governments to provide clean water that can meet people’s expectations in both quality and quantity. One way to combat the depletion of shallow wells is to drill deeper aquifers; the main disadvantage of this approach is the lower quality of the obtained water, and therefore need for a more complicated treatment process is required [1, 2]. There is a fact that around 40% of the global population is experiencing severe freshwater shortages, and this is expected to increase due to population growth. The limitation of available fresh water around the globe has caused numerous problems, and the existing water resources cannot meet potable, irrigation, and industrial needs [3, 4].

Recent advances and development in available technologies have led to a revolution in construction methods. A good example could be the application of higher-capacity dams and power stations or using other water purification techniques like reverse osmosis (RO).

RO is a reputable membrane-based water treatment manner that has been commercialized since the 1960s. RO depends on outside pressure to defeat the osmotic pressure gradient across the membranes. The semi-permeable membranes used in ROs, allow the water molecules to transmit through the pores but trap the more considerable salt molecules, thereby purifying water as it passes via the membrane [5], in other words, the wide applications of RO in water treatment plants are due to their high capability, versatility, and efficiency in removing multi-types of impurities from water [6].

In the case of water distribution systems, Life Cycle Assessment (LCA) can assess the environmental impacts of the system, from the extraction of raw materials to the disposal of the system at the end of its life. The methodology used in LCA involves a systematic evaluation of the environmental impacts of a product, process, or system throughout its entire life cycle. The life cycle includes all stages, from the extraction of raw materials, through production and use, to disposal or recycling.

In the context of RO units in water treatment plants, LCA can be used to evaluate the environmental impacts of the entire water treatment process, including the production of chemicals and materials used in the process, the energy consumption of the treatment plant, and the disposal of waste products. By conducting an LCA, it is possible to identify opportunities for improving the environmental performance of the water treatment process and making more informed decisions about the design and operation of the treatment plant [7].

The semi-permeable membrane used in RO can remove ions, unwanted molecules, and larger particles from drinking water [8]. But, one of the determined concerns in water desalination processes is the climate-changing by greenhouse effects [9]. The CO_2_ equivalent pollution emitted from the RO units can be forecasted using the provided fossil fuel scenarios of RO processes capacity [10, 11]. In addition, in the desalination by RO, all sections of the pumping system, the pretreatment processes, and providing pressure in the RO system are considered energy-consuming. High energy costs contain the environmental impact on energy generation, where GHGs are emitted [12].

Both energy and water are inseparable from economic and social development since energy production requires water, and water attainment, allocation, and consumption stages are accompanied by energy usage [13]. Water treatment plants are no exception from carbon footprint (CF) and CF dependent on embodied energy consumption in various water purification processes [14]. In recent years, high amounts of GHGs (CO_2_ equivalent) have been released, and this is due to the massive use of fossil fuels to meet energy needs. As a result, methods with lower carbon emissions and water purification become socially and politically desirable. Based on the literature reviews, the definition of CF can abbreviate as “A direct measure of greenhouse gas emissions (expressed in tons of carbon dioxide [CO_2_e]) caused by a defined activity. At a minimum, this measurement includes emissions resulting from activities within the control or ownership of the emitter and indirect emissions of the consumption of purchased electricity” [15, 16].

CF in water treatment systems can be measured via both direct and indirect emissions from the treatment processes. Fossil fuel’s utilization to provide energy in current construction and operation processes of water is an example of direct emissions, and material generation in the infrastructures such as pipes, pumps, or others are some examples of indirect emissions in treatment plants [1].

In the last few decades, the proposed approaches to reduce the amounts of used fossil fuels were not effective in the released GHGs emissions from the RO processes, and an urgent need for substituting renewable energy sources was felt, meaning that more efficient renewable energy facilities and more relevant infrastructures are desperately needed to diminish the magnitude of CO_2_e emissions regardless of RO capacity scenarios. If future demands for RO units increase with population growth, it seems this technology has a significant contribution to the emission of greenhouse gases [10].

Several studies focused on reverse osmosis or carbon footprint [17–20]. For example, Missimer et al. [21] surveyed the Sea Water Reverse Osmosis (SWRO) feed quality improvement that demonstrated the biopolymers had the highest removal rate by the Humic substances, building blocks, the light organics, and exclusion over 90% of the bacterial growth from the raw seawater. Also, Felices et al. [8] understood which one of the proper technology for desalination is reverse osmosis, regarding the several phases and energy recovery dimensions and possibilities available along the process. Moreover, the latest produced membranes and employing the most advanced technologies are essential to obtain the maximum desirable efficiency. Since the RO process has been proven to provide an acceptable amount of desalinated water, modifying the proposed technologies offers the safest possible mean for desalination [22].

## Objective

The economic and social development of societies is not possible without the presence of energy and water resources. While many countries have established engineering structures for water treatment, just a few investigations have been done about the carbon footprint in water resources projects. Two particular concerns have been raised about RO in recent decades. The foremost concern is desalination energy usage, which emits high amounts of GHGs. A further concern is the issue of biofouling which leads to increased energy consumption and frequency of chemical cleaning, increases carbon emissions and leads to difficulty in waste disposal [23]. Regarding the possible effects of reverse osmosis on the environment [24], as a result, the drinking water treatment plants equipped with reverse osmosis units will be studied to investigate their GHGs emissions and, a meta-analysis will be used if possible.

### The research question

In the proposed study, a well-defined research question is conducted based on PECO approach. In this regard, the main question is; “What is the carbon footprint of reverse osmosis in water treatment plants?” The sub-components of this question are; water treatment plants/water (with drinking purposes)/water purification as Population, RO/reverse osmosis/desalination/life cycle assessment/LCA as the Exposure water treatment plants without RO is the Comparator, and Carbon emissions/Carbon footprint/CO_2_/GHG emission are the chosen outcomes.

## Method

This text describes a systematic review protocol that adheres to the Collaboration for Environmental Evidence (CEE) guidelines and meets the ROSES reporting standards (Additional file 1). This systematic review protocol is registered in the International Prospective Register of Systematic Reviews (PROSPERO) database, and the registration number is CRD42022327572.

Identifying the system boundaries is a substantial step in conducting a systematic review. The boundaries help to define the scope of the review and ensure that it is focused and relevant.

Carbon footprints are research methods that examine the entire life cycle of a product, from raw materials to packaging, transportation, sales, and customer disposal or recycling. The difference between LCA and carbon footprint relates to impact categories. The carbon footprint is focused on a series of environmental impacts, namely, the total GHGs in which carbon dioxide (CO_2_), methane (CH_4_), and dinitrogen monoxide (N_2_O) are expressed in kilograms of CO_2_ equivalent. Therefore, all greenhouse gases can be presented as a single number and their total emissions multiplied by the global warming potential. Meanwhile, LCA considers more impact categories such as human health, ecosystem quality, and resources. However, when reviewing and comparing LCA results, several challenges can be encountered, including; different studies may use different methodologies, making it difficult to compare results, variability in production systems, the limited number of studies, lack of standardization, and inclusion of other dimensions of sustainability. While LCA focuses on environmental aspects, there is a growing call for more holistic sustainability evaluations that include economic and social dimensions [25].

### Search strategy

After discussion with members of the team and clarification of the main idea of the study based on PECO, the search for literature was divided into two sections;

In the first section, the search will be limited to Scopus, Science Direct, EMBASE, and PubMed databases (Additional file 2). For this stage, the keywords such as water, “water treatment plants”, “water purification”, “reverse osmosis”, RO, desalination, “Life Cycle assessment”, LCA, “carbon emission”, “carbon dioxide emission”, “CO_2_ emission”, “carbon footprint” and “GHGs emission” will be used. These keywords could have been written in the different sections of the text, and the condition for using them in this article was their relevance to the aim of this study. Table 1 has shown the search string in databases. The search strategy was adjusted to the instructions of each database. In addition, more than 10% of the bibliography and references checking proceeded after the full-text screening. Also, the operators such as AND/OR will be used too. The “OR” operator will be used for the deployment of synonyms and the operator of “AND” can reduce unrelated articles. The alarm of databases will be also turned on to send the titles of the latest articles to the e-mail of one of the authors.

**Table 1:** Search methodology in databases

Component	Search string
Population	Water OR “water treatment plant” OR “water purification”
AND	
Intervention/exposure	“Reverse osmosis” OR “RO” OR desalination OR “life cycle assessment” OR LCA
AND	
Outcome	“Carbon footprint” OR “carbon emission*” OR “greenhouse gas emission*” OR “CO_2_ emission*” OR “carbon dioxide” OR “CO_2_”

### Language

The initial search was done in Persian and English, but no relevant study was found in Persian. Since most of the universal surveys are done in English and getting access to the full text of articles in this language is more likely, the main search will be done in English. Also, key regional languages will be conducted for articles that have no English title or abstract [26].

### Duration

No date limitation will be applied in this study.

### Publication databases

The search process for finding the relevant articles will be done using the following databases:

Scopus; https://www.scopus.com/
Science Direct; https://www.sciencedirect.com/
EMBASE; https://www.embase.com
PubMed; https://pubmed.ncbi.nlm.nih.gov/

### Internet searches and specialist searches

In the second section, the search with the same keywords will be done on websites, Google, Google Scholar, grey works of literature, national and international databases, and academics. We assume that in addition to prevalent sites, some NGOs/GOVs also publish information about the effects of greenhouse gas emissions in water purification processes or have activities in this field. Therefore, these reports will also be examined. If more information is needed, we will contact the author of the research. Significantly, we will use the *Publish* or *Perish* software, and the top 1000 relevant results will be selected. The following databases and websites will use for specialist searches.

- Greenhouse gas protocol (https://ghgprotocol.org/)
- UNESCO, unesdoc (https://unesdoc.unesco.org/ark:/48223/pf0000217181)
- The World Bank (https://data.worldbank.org/indicator/EN.ATM.CO2E.KT?**)**
- Google Scholar (https://scholar.google.com/)
- The Intergovernmental Panel on Climate Change (IPCC) (https://www.msci.com/)
- Open Access Theses and Dissertations (https://oatd.org/)
- EPA (https://www.epa.gov/)
- Emissions Database for Global Atmospheric Research (https://edgar.jrc.ec.europa.eu/)
- The Networked Digital Library of Theses and Dissertations (https://ndltd.org/)
- Proquest Theses and Dissertations (https://www.proquest.com/index?selectids=pqdt)
- Wikipedia (https://en.m.wikipedia.org/wiki/Carbon_footprinthttps://en.wikipedia.org/wiki/Desalination)
- International Desalination Association and Global Water Intelligence (https://idadesal.org/)
- Environmental Evidence (https://environmentalevidencejournal.biomedcentral.com/)
- Carbon Leadership Forum (https://carbonleadershipforum.org/)
- The Guardian (https://www.theguardian.com/international)
- OXFAM International (https://www.oxfam.org/)

### Supplemental research and updating

For supplementary research, related articles will be used that are suggested by each chosen essay. Also, citations to articles will be used. In addition, the reference of all the records included will be checked so we do not miss any relevant articles. If it takes more than a year from the search to the publication of results, the search will be updated.

### Assessing research comprehensiveness

For appraising the comprehensiveness of the search we followed the method of Bennett et al. [27]. An independent test set [19, 28–36] was designed and includes ten articles, found in databases, that had the most citations. Then, the eligible references cited in the text of the included articles were used to evaluate the comprehensiveness of the search. If all eligible articles are included, it means that the prepared collection is sufficiently comprehensive. Otherwise, the search method will be updated.

### Article screening and study eligibility criteria

#### Screening process

After a comprehensive search according to the mentioned strategy, the studies found will be collected, and the articles will be reviewed by *Thomson Reuters EndNote X8.1* software. Based on the initial search, it will assume that 6500 records will be identified at this stage, and 5000 records will remain when duplicate records are removed. In the next step, the title and abstract of the research will be examined by two authors in *Rayyan* (https://www.rayyan.ai/). On this website, the authors can define inclusion and exclusion terms, exclusion reasons, add labels to records, justify the language and publishing type, the journal, and years of separation. In the next step, while reading the full text of the articles, the required information will be extracted based on PICO/PECO and collected in a suitable table inside Excel. This information includes the type of water source and the aim of consumption (population), the technology of desalination (intervention/exposure), the amount of emission and how to measure that (outcome), the amount of water treatment per time, type of energy consumption, place and time of the study. Eligibility criteria will be independently applied by more than one reviewer, ideally to all articles screened at the title, abstract, and full-text stages. The reasons for removing each excluded article or study after screening will be mentioned in additional files. The authors will expect that 300 appropriate records will remain at the end of the screening. Exclusion criteria comprise unrelated, duplicated, unavailable full texts, abstract-only papers, and pamphlets.

#### Eligibility criteria

**Fig. 1:**
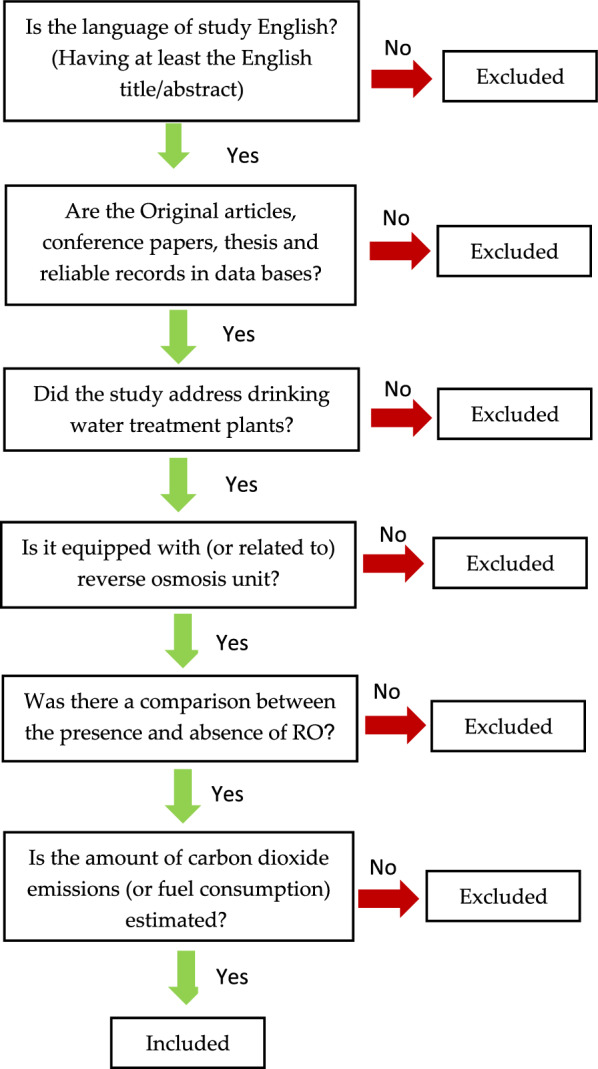
Flow diagram of article screening and study eligibility criteria

Eligibility criteria will be based on the PICO/PECO approach, study setup, and date (Table 2). These criteria will be stated in advance to refrain the researcher from bias. There would be articles that contain information helping to answer the research question. But the most considerable matter will be that these articles should be clear and have sufficient data, including positive or negative reports to answer the question. After that, the full text of the study will be retrieved based on eligibility paragons by two other authors. In the screening stage, 10% of the articles (at least 50) will be screened by two reviewers to measure the agreement between the reviewers. We will use the Kappa test to determine the agreement between the reviewers. Cohen’s kappa coefficient was set at 0.81 for significant agreement [24]. In case of disagreement, researchers will discuss, or the opinion of the third impartial reviewer will be used. About language, to include the records in the current study, they must be in English, but in order not to neglect studies that do not have English abstracts/titles, key regional languages will be reviewed. Also, studies must be original and reliable, and review studies will be excluded. The latter condition for entering the review is the RO unit application in drinking water treatment plants. Since these treatment units have many uses, drinking water treatment plants were considered. Also, the studies should have calculated and stated the amount of greenhouse gas emissions from these units. All aspects of searching will be made based on Fig. 1.

**Table 2:** Eligibility criteria

Criteria	Inclusion	Exclusion
Types of study	Original articles, conference papers, and thesis	Review articles, books and any, letters to editor, record that has not efficient data
Language	Studies that were in English language included	Studies that are in languages other than English that could make translating bias
Population	Water, water treatment plant or water purification that it equipped with a reverse osmosis unit with drinking aims	Water for non-drinking purposes or treatment plants that are not equipped with reverse osmosis units
Exposure	Reverse osmosis or RO, desalination, life cycle assessment, LCA	Other purification methods that RO has not the main role
Comparator	Drinking water treatment plants with RO in comparison with other technologies	Failure to compare with RO
Outcome	Carbon footprint, carbon emission, greenhouse gas emission or CO_2_ emission	No calculation or no expression of the amount of emission
Duration	There is no limiting time	–
Study area	Universal	–

#### Study validity assessment

Bias or inaccuracy is referred to deviation from the truth or a systematic error in the study’s findings and includes the inference made in the study. The validity assessment will be done based on Collaboration for Environmental Evidence Critical Appraisal Tool Version 0.3 (Additional file 3). This tool was developed for evaluating the “risk of bias” (or threats to internal validity) of primary studies assessing the effectiveness of interventions or impacts of exposures in environmental management. The risk of bias spectrum provided in this tool is categorized as low, medium, and high. It is theoretically possible that study findings are not biased if sources of bias are eliminated sufficiently in any study layout or if approaches are justified on a case-by-case basis based on addressed causal structure and available data. However, this tool does not assume that differences in study designs alone affect the accuracy of study findings. It presumes that effect estimates may be biased if sources of bias are not controlled for or not taken into account. Although the distinction between bias and imprecision should be kept intelligibly in mind when applying this tool, biases in real-world data cannot often be quantified, and thus perfectly distinguishing between them is not possible unless correct values are known. It is why the concept of risk of bias (a measure of threats to internal validity) was developed, and it is now widely applied to evaluate how susceptible studies are to biases in the healthcare sector rather than trying to quantify biases. By using this tool, study findings will always have risks of bias since it is not often possible to prove that there are no biases in results. Having risks of bias does not mean that the findings are biased but there are always possibilities for findings being biased to some extent [37]. When dealing with carbon footprint data, it's main to consider the possibility of a skewed distribution. To meet the assumptions of statistical models used in the meta-analysis it may be necessary to transform the data in several ways. One common approach to dealing with skewed data is applying a logarithmic transformation that can help normalize the data distribution and make it more suitable for utilization in statistical models. Other possible changes include square root or inverse transformations, although these may not be as effective as a logarithmic transformation [38, 39].

In summary measures and pooling effect sizes across studies, it's important to consider the heterogeneity of the data and the potential for differences in study design, measurement methods, and other factors that could impact the results. One approach to address this heterogeneity is to use a random effects model, which accounts for both within-study and between-study variability.

#### Data coding and extraction strategy

After the article's full-text evaluation, the following raw data will be collected and categorized in an Excel spreadsheet (Additional file 4). This information includes the type of untreated water source, the purpose of use (drinking), covered population and the target society, the volume of water to be treated, reverse osmosis capacity, module working period, type of fuel consumed, amount of fuel consumption, and the method of calculating carbon dioxide emissions. Data ensuring for assessing the accuracy of extraction will be performed by two authors. In case of disagreement between them, the opinion of the rest authors will be used.

#### Potential effect modifiers/reasons for heterogeneity

Meta-analyses should assess heterogeneity, which may be defined as the variation entity in the true effect sizes underlying different studies. This assessment can be achieved by performing a statistical test for heterogeneity, quantifying its amount and effects, or a mixture of these [40]. The main factor that can cause heterogeneity in the results is the location of the reverse osmosis modules. RO devices are widely used around the gulfs and beaches, but evidence suggests that this equipment can also be used in different centers [41]. Therefore, the used source of water and its quality can lead to changes in fuel consumption, the pattern of reversing filters, the use of chemicals, etc., and can lead to a change in global emissions. The next factor that will cause heterogeneity is the measurement method of environmental footprint. One of the principles and fundamental methods to study the environmental effects of a procedure, system, or product will be the application of LCA.

LCA is a method widely used to assess the environmental impact of products and processes throughout their entire life cycle. A product, process, or activity's entire life cycle can be identified, quantified, and assessed using the LCA methodology. From beginning to end, it considers how energy and materials are used and released into the environment (such as during the extraction, production, use, and disposal of raw materials). The LCA essentially entails applying mass and energy balances to the system under study and assessing any potential environmental effects related to inputs and outputs. In order to better guide future research, it is helpful to pinpoint “hot spots” of potential environmental impacts and to set baselines [8, 42].

In the case of LCA, the functional unit, system boundaries, data sources, and impact assessment methods are key methodological choices that can influence the results. The functional unit measurement unit that used to define the scope and boundaries of the LCA study. It is important to choose a functional unit that accurately represents the product or process being evaluated and allows for meaningful comparisons between different products or processes. System boundaries define the scope of the LCA study and determine which processes and activities are included in the assessment [43]. Electricity consumption, input and output flows of material (mainly chemicals) and energy resources (conventional energy sources). The energy needed for regulating the pump pressure was taken into account but reject water was not considered, since it can be reused. Material flows in construction and operational phase, disposal flows in dismantling phase, transportation of construction material, membranes, chemicals and waste materials, concentrate disposal, pretreatment system and water distribution system were assumed as system boundaries [44, 45].

#### Data synthesis and presentation

Heterogeneity should be expected in a meta-analysis: it would be unexpected if multiple surveys were conducted by different teams in different locations with numerous approaches and all ended up with similar underlying parameter estimates. We are faced with the question of whether there is an “acceptable” degree of heterogeneity because heterogeneity is inevitable in a meta-analysis. Then the challenge is to decide on the most appropriate way to analyze heterogeneous studies, and this depends on the aims of the synthesis and, to some extent, the directions and magnitudes of observed effects. It may involve a random-effects meta-analysis, where heterogeneity is assumed to be of a particular form (often, but not necessarily, normally distributed), or it may involve combining covariates at the study level [46].

The meta-analysis has three major aims: test the homogeneity of studies, receive a global index about the effect magnitude of the surveyed relation, and determine possible variables or elements moderating the results obtained if there is heterogeneity among studies. Two sources of variability explain the heterogeneity in a set of studies in a meta-analysis. One is the variability due to sampling error, also named within-study variability. The other origin of heterogeneity is the between-studies variability, which can occur when there is true heterogeneity in a meta-analysis among the population effect sizes estimated by the separate studies. The Cochrane’s *Q* test is the normal way for assessing later heterogeneity and another way is estimating variance with *t*^2^. Assuming a random-effects demonstration, the between-studies fluctuation reflects how much the correct population impact sizes assessed within the single consideration of a meta-examination contrast [47].

In the context of carbon footprint, “effect size” refers to the magnitude of the impact that a particular variable has on the carbon footprint of reverse osmosis units in water treatment plants. Effect size is typically measured using a statistical technique called regression analysis, which involves evaluating the relationship between a dependent variable (such as carbon footprint) and one or more independent variables (such as energy consumption or water recovery rate). In the case of reverse osmosis units, effect size can be used to identify the variables that have the greatest impact on GHGs emissions and to prioritize strategies for reducing carbon emissions. For example, if the effect size of energy consumption is found to be particularly large, then strategies for reducing energy consumption (such as using more efficient pumps or optimizing the operation of the reverse osmosis unit) may be prioritized over other strategies [48]. In the context of reverse osmosis, LCA can assess not only the direct carbon emissions associated with the operation of the system but also the indirect effects. Indirect effects refer to the upstream and downstream processes that contribute to the carbon footprint of reverse osmosis, such as the production and transportation of raw materials, energy generation, and waste management. By conducting an LCA, it is possible to quantify and analyze the indirect effects of carbon footprint in reverse osmosis. This allows for a more comprehensive understanding of the environmental impact of the entire system and helps identify areas where improvements can be made to reduce carbon emissions throughout the life cycle [34, 49].

For this study, tables and figures will be used to summarize the obtained results followed by an interpretation and discussion. If some of the chosen articles meet the requirements for quantitative synthesis, quantitative data analysis will be performed for them. For the studies that have sufficient and appropriate data, meta-analysis will be used to analyze them. Also, the studies that have incomplete or missing data will be not included in the meta-analysis. If there is heterogeneity between the studies included in the analysis, we will use appropriate test to assess heterogeneity. If there is high heterogeneity, subgroup analysis or meta-regression will be used to determine the source of heterogeneity. To check the publication bias, a funnel plot tool will be used by which the comparison of the study effect size with the standard error may be used. If it is not possible to use the funnel plot, the Egger test will be used to determine the publication bias. In the review report, full details of meta-analysis methods will be presented.

## Supplementary Information

**Additional file 1: **ROSES file

**Additional file 2: **Search string example

**Additional file 3: **Environmental Evidence Critical Appraisal Tool Version 0.3

**Additional file 4: **Data coding and extraction strategy

## Data Availability

Not applicable.

## Abbreviations

GHGs: Greenhouse gases
RO: Reverse osmosis
CCE: Collaboration for environmental evidence
CO_2_: Carbon dioxide
CF: Carbon footprint
WHO: World Health Organization
SWRO: Sea water reverse osmosis
PRISMA-P: Systematic review and meta-analysis protocols
PROSPERO: International prospective register of systematic reviews
LCA: Life cycle assessment
