# Supplementary material for: What is the carbon footprint of reverse osmosis in water treatment plants? A systematic review protocol
Source: Environ Evid. 2023 Nov 14;12:23. doi: 10.1186/s13750-023-00316-z (PMC11378811; doi:10.1186/s13750-023-00316-z)
Supplement: Supplementary file 2 — Additional file 2: Search string example [file 13750_2023_316_MOESM2_ESM.docx]

In the first section, the search will be limited to Scopus, Science Direct, EMBASE, and PubMed databases. For this stage, the keywords such as water, "water treatment plants", "water purification", "reverse osmosis", RO, desalination, "carbon emission", "carbon dioxide emission"/"CO_2_ emission", "carbon footprint" and "GHG emission" will be used. These keywords could have been written in the different sections of the text, and the condition for using them in this article was their relevance to the aim of this study.

**Below, there is a table that shows search strings in PubMed databases.**

| **Database** | **Population** |  | **Exposure/**  **Intervention** |  | **Outcome** |
| --- | --- | --- | --- | --- | --- |
| PubMed | ("Water (Purification"[Mesh]) | **AND** | ("Reverse osmosis") | **AND** | ("Carbon Footprint"[Mesh]) |
|  | **OR** |  | **OR** |  | **OR** |
|  | ("Water"[Mesh]) |  | (RO) |  | ("carbon emission") |
|  | **OR** |  | **OR** |  | **OR** |
|  | ("water treatment plant") |  | (desalination) |  | (greenhouse gas emission) |
|  |  |  |  |  | **OR** |
|  |  |  |  |  | ("CO_2_ emission") |
|  |  |  |  |  | **OR** |
|  |  |  |  |  | ("carbon dioxide emission") |
