# Supplementary material for: What is the carbon footprint of reverse osmosis in water treatment plants? A systematic review protocol
Source: Environ Evid. 2023 Nov 14;12:23. doi: 10.1186/s13750-023-00316-z (PMC11378811; doi:10.1186/s13750-023-00316-z)
Supplement: Supplementary file 3 — Additional file 3: Environmental Evidence Critical Appraisal Tool Version 0.3 [file 13750_2023_316_MOESM3_ESM.docx]

**README**

This file presents a list of benchmark articles (test set) of known relevance to **" What is the Carbon Footprint of Reverse Osmosis in Water Treatment Plants? A systematic review protocol"**

These should be returned, following a search using the search string, to determine the comprehensiveness of the search.

| Study title | Year |
| --- | --- |
| Energy and Air Emission Effects of Water Supply | 2009 |
| A comparative life cycle assessment of process water treatment technologies at the Secunda industrial complex, South Africa | 2012 |
| Environmental life cycle assessment of seawater reverse osmosis desalination plant powered by renewable energy | 2014 |
| Environmental and economic assessment of beach well intake versus open intake for seawater reverse osmosis desalination | 2015 |
| Comparative life cycle assessment of end-of-life options for reverse osmosis membranes | 2015 |
| Life Cycle Assessment of RO Water Desalination System Powered by Different Electricity Generation Alternatives | 2017 |
| Life Cycle Assessment of a seawater reverse osmosis plant powered by a hybrid energy system (fossil fuel and waste to energy) | 2021 |
| Greenhouse Gas Management Plan | 2022 |
| Carbon footprint of drinking water over treatment plant life span (2025–2075) is probably dominated by construction phase | 2022 |
| Life Cycle Assessment of Upgrade Options to Improve Nutrient Removal for the City of Santa Fe, NM, Paseo Real Wastewater Treatment Plant | 2023 |

**Table 2. The test set for search comprehensiveness assessing**

**References**
